# Supplementary material for: Multi‐dataset identification of innovative feature genes and molecular mechanisms in keratoconus
Source: J Cell Mol Med. 2024 Sep 19;28(18):e70079. doi: 10.1111/jcmm.70079 (PMC11412914; doi:10.1111/jcmm.70079)
Supplement: Supplementary file 2 — Material S2. [file JCMM-28-e70079-s002.zip › 7.GSEA analysis/6.SNORA74B/KEGG_PENTOSE_AND_GLUCURONATE_INTERCONVERSIONS.html]

Details for gene set KEGG\_PENTOSE\_AND\_GLUCURONATE\_INTERCONVERSIONS[GSEA]

|  || Dataset | SNORA74B.group.cls#h\_versus\_l |
| Phenotype | group.cls#h\_versus\_l |
| Upregulated in class | h |
| GeneSet | KEGG\_PENTOSE\_AND\_GLUCURONATE\_INTERCONVERSIONS |
| Enrichment Score (ES) | 0.35765475 |
| Normalized Enrichment Score (NES) | 0.96195704 |
| Nominal p-value | 0.4969819 |
| FDR q-value | 1.0 |
| FWER p-Value | 1.0 |
Table: GSEA Results Summary

  

Fig 1: Enrichment plot: KEGG\_PENTOSE\_AND\_GLUCURONATE\_INTERCONVERSIONS      
 Profile of the Running ES Score & Positions of GeneSet Members on the Rank Ordered List

  

| SYMBOL | TITLE | RANK IN GENE LIST | RANK METRIC SCORE | RUNNING ES | CORE ENRICHMENT || 1 | UGT1A8 | na | 321 | 0.680 | 0.2178 | Yes |
| 2 | UGT1A6 | na | 2066 | 0.366 | 0.2896 | Yes |
| 3 | UGT2B4 | na | 3661 | 0.272 | 0.3345 | Yes |
| 4 | UGT2B11 | na | 6088 | 0.190 | 0.3279 | Yes |
| 5 | UGT2B28 | na | 7492 | 0.149 | 0.3372 | Yes |
| 6 | RPEL1 | na | 8559 | 0.123 | 0.3476 | Yes |
| 7 | UGT2B10 | na | 9771 | 0.097 | 0.3451 | Yes |
| 8 | DCXR | na | 10337 | 0.086 | 0.3577 | Yes |
| 9 | UGT2A3 | na | 13387 | 0.043 | 0.2841 | No |
| 10 | RPE | na | 18760 | -0.014 | 0.1342 | No |
| 11 | CRYL1 | na | 18810 | -0.015 | 0.1378 | No |
| 12 | GUSB | na | 19513 | -0.023 | 0.1252 | No |
| 13 | DHDH | na | 19938 | -0.028 | 0.1223 | No |
| 14 | UGP2 | na | 20473 | -0.034 | 0.1182 | No |
| 15 | UGT2A1 | na | 21014 | -0.040 | 0.1160 | No |
| 16 | UGDH | na | 21842 | -0.050 | 0.1088 | No |
| 17 | XYLB | na | 23634 | -0.075 | 0.0823 | No |
| 18 | UGT2B15 | na | 25363 | -0.105 | 0.0675 | No |
| 19 | AKR1B1 | na | 31422 | -0.290 | -0.0103 | No |
| 20 | UGT2B7 | na | 31877 | -0.316 | 0.0821 | No |
Table: GSEA details [plain text format]

  

Fig 2: KEGG\_PENTOSE\_AND\_GLUCURONATE\_INTERCONVERSIONS      
 Blue-Pink O' Gram in the Space of the Analyzed GeneSet

  

Fig 3: KEGG\_PENTOSE\_AND\_GLUCURONATE\_INTERCONVERSIONS: Random ES distribution      
 Gene set null distribution of ES for **KEGG\_PENTOSE\_AND\_GLUCURONATE\_INTERCONVERSIONS**
